# Structural variation of the coding and non-coding human pharmacogenome

**DOI:** 10.1038/s41525-023-00371-y

**Published:** 2023-09-08

**Authors:** Roman Tremmel, Yitian Zhou, Matthias Schwab, Volker M. Lauschke

**Affiliations:** 1 Dr Margarete Fischer-Bosch Institute of Clinical Pharmacology, Stuttgart, Germany; 2 University Tübingen, Tübingen, Germany; 3 Department of Physiology and Pharmacology, Karolinska Institutet, Stockholm, Sweden; 4 Departments of Clinical Pharmacology and Pharmacy and Biochemistry, University Tübingen, Tübingen, Germany

**Keywords:** Molecular medicine, Predictive markers, Functional genomics

## Abstract

Genetic variants in drug targets and genes encoding factors involved in drug absorption, distribution, metabolism and excretion (ADME) can have pronounced impacts on drug pharmacokinetics, response, and toxicity. While the landscape of genetic variability at the level of single nucleotide variants (SNVs) has been extensively studied in these pharmacogenetic loci, their structural variation is only poorly understood. Thus, we systematically analyzed the genetic structural variability across 908 pharmacogenes (344 ADME genes and 564 drug targets) based on publicly available whole genome sequencing data from 10,847 unrelated individuals. Overall, we extracted 14,984 distinct structural variants (SVs) ranging in size from 50 bp to 106 Mb. Each individual harbored on average 10.3 and 1.5 SVs with putative functional effects that affected the coding regions of ADME genes and drug targets, respectively. In addition, by cross-referencing pharmacogenomic SVs with experimentally determined binding data of 224 transcription factors across 130 cell types, we identified 1276 non-coding SVs that overlapped with gene regulatory elements. Based on these data, we estimate that non-coding structural variants account for 22% of the genetically encoded pharmacogenomic variability. Combined, these analyses provide the first comprehensive map of structural variability across pharmacogenes, derive estimates for the functional impact of non-coding SVs and incentivize the incorporation of structural genomic data into personalized drug response predictions.

## Introduction

Inter-individual variability in drug response has long been recognized as a major problem in pharmacological treatment. Overall, it is estimated that around 50% of patients experience a lack of efficacy or adverse drug reactions (ADRs), contributing to considerable patient morbidity and mortality^1^. In addition to posing a significant burden on the healthcare system, lack of drug efficacy and ADRs are major hurdles to drug development. More than 80% of candidate drugs fail in clinical trials and around 32% of FDA-approved therapeutics are affected by post-market safety events^2,3^. Mechanistically, variable drug responses can stem from variability in drug disposition or altered pharmacodynamics.

Heritable factors play an important role in differential drug response and genetic variability, including variations in genes modulating drug pharmacokinetics as well as drug targets, explain approximately 20–30% of inter-individual phenotypic differences^4^. Among these, single nucleotide variants (SNVs) have been extensively studied as biomarkers to predict drug efficacy and ADRs. A multitude of such variants in genes involved in drug absorption, distribution, metabolism and excretion (ADME) has been included in the pharmacogenomic guidelines to individualize pharmacological treatment based on patient genotypes^5–7^. Comparatively less is known about the functional effects of pharmacogenetic drug target variability. While the landscape of SNVs in drug targets has been systematically analyzed^8^ and elegant recent studies demonstrated striking effects of SNVs on intracellular signal transduction and drug action^9,10^, more evidence is required to enable the translation of such variations into clinical recommendations.

In contrast to SNVs, structural variations (SVs), defined as genomic deletions, duplications, insertions, inversions and other complex rearrangements that affect >50 bp, are substantially less studied^11,12^. While the total number of SVs per human genome is around two orders of magnitude lower than for SNVs (34,000 SVs compared to 3 million SNVs), SVs affect 3.4 times more nucleotides in both coding and non-coding regions of the genome^13^ and constitute important contributors to human phenotypes^14–16^. Copy number variations (CNVs) in some ADME genes are well described^17,18^, whereas the structural variability of human drug targets has not been systematically analyzed. Furthermore, comprehensive analyses of non-coding structural variability in pharmacogenes have not been presented. Here, we systematically profiled the landscape of structural variability across 908 pharmacogenes (344 ADME genes and 564 drug targets) based on whole genome sequencing (WGS) data from 10,847 unrelated individuals^19^. Our analyses refine previous SV frequency estimates and, by integrating structural data with experimentally determined transcription factor binding site (TFBS) information, identify a catalog of 1276 SVs that impact pharmacogenetic regulatory elements.

## Results

### The structural variome in genes involved in drug disposition and drug targets

**Fig. 1:**
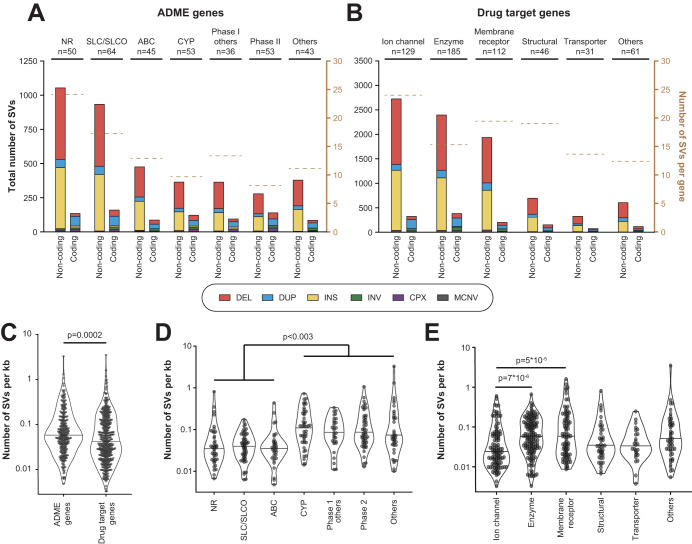
Overview of structural variability in the human pharmacogenome. Number of structural variants (SVs) in different classes of genes that are involved in drug absorption, distribution, metabolism and excretion (ADME; **A**) as well as drug target genes (**B**). **C** The number of SVs per kilo base (kb) gene length differ significantly between ADME genes and drug targets. **D** Among ADME genes, CYPs, as well as phase 1 and phase 2 enzymes harbor significantly more SVs than nuclear receptors and transporters. **E** Among drug target genes, ion channels were significantly less variable than enzymes and membrane receptors. NR nuclear receptors, CYP cytochrome P450s, SLC solute carrier transporters, ABC ATP binding cassette transporters, CPX complex rearrangement, DEL deletion, DUP duplication, INS insertion, INV inversion, MCNV multi-copy number variations.

We first analyzed the structural variability of 344 genes involved in ADME processes. The highest number of SVs was found in nuclear receptors (*n* = 1207; average of 24 SVs per gene) and SLC/SLCO transporters (*n* = 1112; average of 17 SVs per gene), whereas SV numbers in phase II enzymes were around 3-fold lower (*n* = 437; 8 SVs per gene; Fig. 1A). Additionally, we analyzed the structural variome in 564 genes encoding the therapeutic targets of 1578 clinically approved drugs. Most SVs were identified in ion channels (*n* = 3112; 24 SVs per gene) and membrane receptors (*n* = 2840; 19 SVs per gene), whereas the variability in transporter targets was markedly lower (*n* = 427; 14 SVs per gene; Fig. 1B). *PTGS2* (*n* = 189), *GPD2* (*n* = 150), *HCN1* (*n* = 145) and *KCND2* (*n* = 145) featured the most SVs whereas 41 pharmacogenes did not harbor any structural variations (Supplementary Table 3). When normalizing for gene length, ADME genes carried significantly more SVs per kilo base than drug targets (Fig. 1C). The higher variability was primarily driven by genes encoding drug metabolizing enzymes (CYPs, as well as other phase 1 and phase 2 enzymes), whereas transporter genes and nuclear receptors were significantly less variable and harbored similar numbers than drug target genes (Fig. 1D, E).

SVs range in size from 50 bp to 106 Mb with a median size of 312 bp (Supplementary Fig. 1A). Drug target SVs were overall significantly shorter than SVs in ADME genes (281 bp vs 321 bp; *p* < 0.0001). The overall largest SVs (106 Mb) was a singleton complex rearrangement of duplications and inversions that affected almost the complete chromosome 10 covering a total of 589 genes, as well as a rare duplication on chromosome 5 that affected the target genes *IL6ST*, *GHR*, *HCN1*, *NDUFAF2*, *NDUFS4*, *PDE4D*, *PTGER4* (28 Mb). The longest deletions affected the GABA receptor cluster encoding *GABRA1*, *GABRA6,* and *GABRG2* on chromosome 5 (6.5 Mb) and the ADME gene *COMT* on chromosome 22 (2.5 Mb). Insertions and deletions had median sizes of 208–618 bp, whereas the average inversions were more than 10,000 times larger with a median size of 30.2 Mb (Supplementary Fig. 1B–G). Furthermore, both ADME and drug target SVs were significantly smaller than SVs in olfactory genes (*p* < 0.0001), which were selected as one of the most polymorphic human gene families due to low selective pressure^20^.

### Functional consequences of coding pharmacogenomic structural variability

**Fig. 2:**
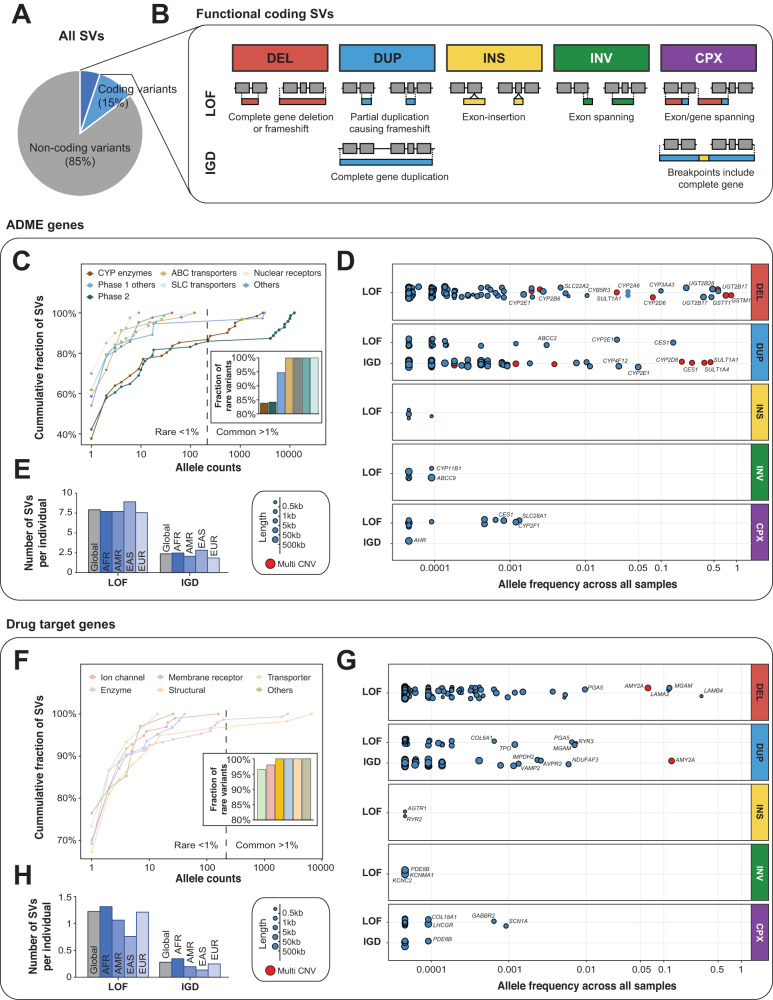
The landscape of functional SVs across the pharmacogenome. **A** Across all identified SVs in pharmacogenes, 15% affected exons (blue) and 85% were non-coding (gray). Of the exonic variations, functional consequences could be inferred for one third (dark blue), whereas the functional consequences of the remainder were unknown (mostly SVs in UTRs or in-frame deletions/duplications). **B** Schematic showing the different SV classes causing loss-of-function (LOF) or increased gene dosage (IGD) of the corresponding gene. **C** Fraction of rare and common SVs for the different ADME gene families. **D** Allele frequencies for structural LOF and IGD variants across ADME genes. The sizes of dots indicate the length of the corresponding SVs. Multi CNVs are indicated in red. **E** Bar plots show the average number of LOF and IGD SVs in ADME genes per individual for the entire dataset (global) and for individual ethnogeographic groups. **F** Fraction of rare and common SVs in the different drug target gene classes. **G** Allele frequencies are shown for structural LOF and IGD variants across drug targets. The sizes of dots indicate the length of the corresponding SVs. Multi CNVs are indicated in red. **H** Bar plots show the number of LOF and IGD SVs in drug target genes per individual for the entire dataset (global) and for individual ethnogeographic groups. CPX complex rearrangement, DEL deletion, DUP duplication, INS insertion, INV inversion, AFR African, AMR admixed Americans, EAS East Asians, EUR Europeans.

Of all 14,984 pharmacogenomic SVs, 2198 impacted gene exons, whereas the remainder affected introns, or non-coding regions up- and downstream of the gene body (Fig. 2A). To interpret SV functionality, we classified deletions spanning coding regions as well as exonic insertions, exon-spanning inversions or partial gene duplications that resulted in frameshifts as LOF SVs (Fig. 2B). In contrast, duplications of the entire gene were considered as increased gene dosage (IGD). While these variations can result in gain-of-function effects, as shown e.g. for *CYP2D6*^21^ and *SULT1A1*^22^, gene duplications in other pharmacogenes, such as *CYP2E1*, resulted in dosage insensitive expression and activity^23^.

All exonic SVs in drug transporters and nuclear receptors with putative functional consequences were rare with MAF < 1%, whereas up to 20% of SVs in genes encoding CYPs (*n* = 9 SVs), other phase I (*n* = 2) or phase II enzymes (*n* = 11) were common (Fig. 2C). LOF SVs with high frequency were identified in *GSTM1* (84.5% deletion frequency), *GSTT1* (71.8% deletion frequency), *UGT2B17* (56% deletion frequency), *UGT2B28* (21.5% deletion frequency) and *CYP2D6* (7.8% deletion frequency; Fig. 2D and Table 1). Similarly, common IGD SVs were found in *SULT1A1* (45.1% duplication frequency), *SULT1A4* (37.2% duplication frequency), *CES1* (25.6% duplication frequency) and *CYP2D6* (18.8% duplication frequency). In aggregate, each individual harbored on average 7.9 LOF and 2.4 IGD SVs in ADME genes, which might contribute to inter-individual differences in response to medications metabolized or transported by the respective gene products (Fig. 2E). Notably, East Asians harbored most (11.7 per individual) and Europeans the least (9.4 per individual) functional coding SVs in ADME genes.

**Table 1:** Common functional coding SVs in pharmacogenes with minor allele frequencies above 1%.

Gene	Type	Function	Length (bp)	Frequency	Drug associations
*ADME genes*					
*GSTM1*	MCNV (DEL)	LOF	19000	84.5%	Cisplatin, nevirapine, azathioprine
*GSTT1*	MCNV (DEL)	LOF	54150	71.8%	Cisplatin, thalidomide, clozapine
*UGT2B17*	MCNV (DEL)	LOF	117000	56%	Ibuprofen, exemestane
*GSTT2B*	DEL	LOF	37153	46.2%	Cisplatin
*SULT1A1*	MCNV (DUP)	IGD	13000	45.1%	Acetaminophen, minoxidil, estrogens
*SULT1A4*	MCNV (DUP)	IGD	50000	37.2%	Salbutamol, acetaminophen, morphine
*CES1*	MCNV (DUP)	IGD	17500	25.6%	Clopidogrel, irinotecan, methylphenidate
*UGT2B28*	DEL	LOF	109294	21.5%	Androgens
*CYP2D6*	MCNV (DUP)	IGD	12202	18.8%	Antidepressants, antipsychotics, opioids, tamoxifen
*CES1*	DUP	LOF	6300	14.4%	Clopidogrel, irinotecan
*CYP3A43*	DEL	LOF	2171	9.9%	Erythromycin, olanzapine
*CYP2D6*	MCNV (DEL)	LOF	12202	7.8%	Antidepressants, antipsychotics, opioids, tamoxifen
*CYP2E1*	DUP	IGD	91684	5%	Acetaminophen, ethanol, anti-tuberculosis drugs
*CYP2A6*	DEL	LOF	30617	3.6%	Nicotine
*CYP4F12*	DUP	IGD	53300	2.8%	Astemizole
*CYP2E1*	DUP	LOF	9600	2.6%	Acetaminophen, ethanol, anti-tuberculosis drugs
*SULT1A1*	MCNV (DEL)	LOF	13000	2.6%	Acetaminophen, minoxidil, estrogens
*CYP2A6*	DUP	IGD	30939	1.1%	Nicotine
*CYB5R3*	DEL	LOF	511	1%	Metoclopramide, lidocaine, dapsone
*UGT2B10*	DUP	IGD	37000	1%	Amitriptyline, olanzapine, nicotine
*Drug target genes*					
*LAMB4*	DEL	LOF	254	30.6%	Ocriplasmin
*AMY2A*	MCNV (DUP)	IGD	165900	12.6%	Acarbose
*MGAM*	DEL	LOF	25985	11.8%	Miglitol, acarbose, voglibose
*LAMA2*	DEL	LOF	835	11.5%	Ocriplasmin
*AMY2A*	MCNV (DEL)	LOF	165900	6.2%	Acarbose

*DEL* deletion, *DUP* duplication, *IGD* increased gene dosage, *LOF* loss of function, *MCNV* multi-allelic copy number variation.

For pharmacodynamic drug targets, more than 95% of all coding SVs were rare with the only exceptions being found in structural genes (laminins) and enzymes (alpha glucosidases; Fig. 2F, G and Table 1). The laminins *LAMA2* and *LAMB4* are targets in the treatment of ocriplasmin vitreomacular adhesion, whereas the amylases *AMY2A* and *MGAM* are targeted by acarbose, voglibose and miglitol for the improvement of postprandial hyperglycemia. Overall, the number of drug target SVs is 5–10 times lower than in ADME genes with each individual harboring a total of 1.2 LOF and 0.3 IGD SVs (Fig. 2H). In contrast to SVs in ADME genes, aggregated SV frequencies differed almost 2-fold between ethnogeographic groups with the lowest numbers of functional SVs across drug targets in East Asians (0.88 per individual) and the highest number in individuals of African ancestry (1.64 per individual).

### Interpreting the functionality of non-coding SVs

**Fig. 3:**
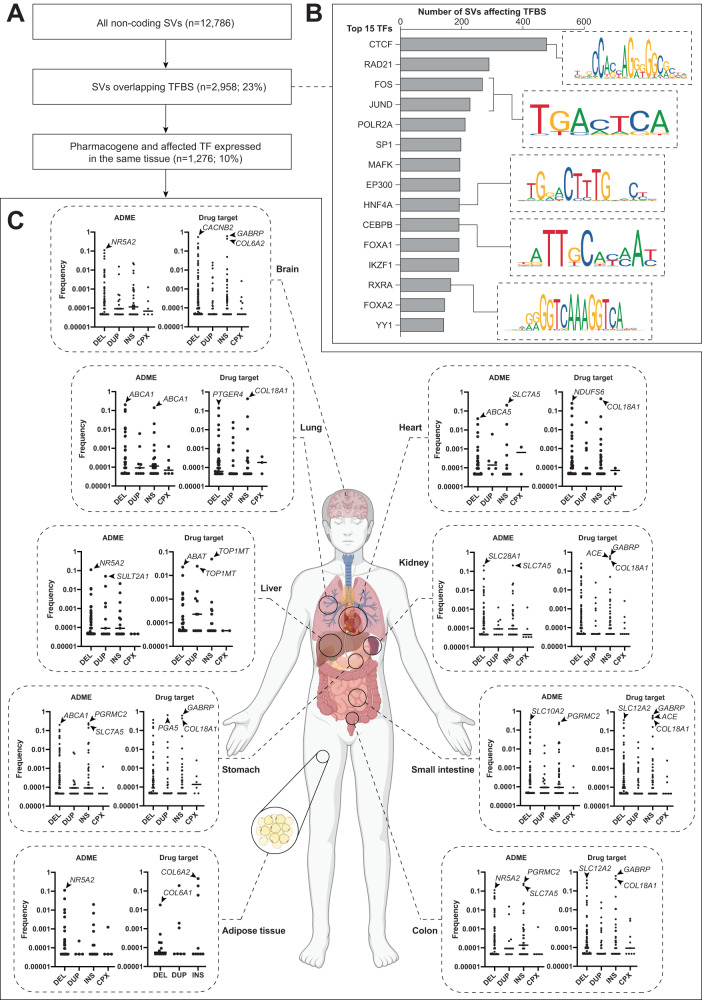
Non-coding SVs overlap with transcription factor binding sites. **A** All identified non-coding SVs were first filtered based on their overlap with transcription factor binding sites (TFBS). Subsequently, those SVs where the impacted pharmacogene and transcription factor (TF) were expressed in the same tissue were taken forward. **B** 15 TFs that are affected by the highest number of SVs. Binding motifs were indicated for selected TFs. **C** Frequencies of SVs for which expression of the corresponding pharmacogene and transcription factor overlap in nine human tissues. SVs with top frequencies were highlighted in both ADME genes and drug targets.

While the consequences of SVs in coding regions have been studied extensively, interpretation of the functional effects of non-coding structural variability, which account for >85% of all pharmogenomic structural variation, has not yet been presented. Here, we inferred functional effects by analyzing the overlap of structural variation with experimentally determined transcription factor binding site (TFBS) data of 224 transcription factors and their expression across 130 cell types and tissues. Of all 12,786 non-coding SVs identified in ADME genes and drug targets, 2958 (23.1%) overlapped with at least one TFBS (Fig. 3A). The most commonly affected binding motifs corresponded to transcription factors with globally important functions, such as CTCF (impacted by 481 SVs), which plays critical roles in genome partitioning and maintenance of the chromosomal architecture, RAD21 (291 SVs), a member of the cohesin complex, and FOS (272 SVs) and JUND (232 SVs), which dimerize to form the AP-1 transcription complex that plays pleiotropic roles in the activation of gene expression (Fig. 3B). Further, various binding sites of key tissue-specific transcription factors were impacted, including HNF4A (affected by 197 SVs), a transcription factor of central importance for hepatopancreatic development and xenobiotic response^24^, and RXRA (affected by 169 SVs), a combinatorial partner that dimerizes with approximately one third of nuclear receptors in human liver^25^.

Since most TFs are not ubiquitously expressed, SVs in their respective TFBSs can only impact the target gene expression in tissues where the respective transcription factor is expressed. We thus analyzed the expression overlap of pharmacogenes (both ADME and drug targets) that harbor SVs affecting TFBSs with the respective transcription factors across nine tissues of major pharmacokinetic or pharmacodynamic importance (Fig. 3C). In total, we identified 1276 non-coding SVs where the affected gene and the respective transcription factor were co-expressed in at least one tissue with each individual carrying an estimated average of 21.7 putatively functional pharmacogenomic SVs (Supplementary Table 4).

Deletions of TFBSs ablate TF activity for the associated gene, which would entail reduced or increased expression in the case of transcriptional activators or repressors, respectively. Inversely, duplication of TFBSs can be expected to have opposite effects. In ADME genes, the highest frequency of such non-coding deletions affecting TFBSs was found in *SLC10A2* (encoding the intestinal transporter ASBT; MAF = 25.9%) where it affected the binding sites of the co-expressed transcription factors CTCF (Table 2). Similarly, deletion of TFBSs of CTCF, RAD21 and SP1 in *SLC28A1* encoding the renal transporter CNT1 was identified in 20% of alleles, and the most common deletion of an hepatic gene was found in hepatic sulfotransferase *SULT2A1* (MAF = 5.4%), affecting TFBSs of CTCF, CHAMP1, ATF2 and CREB1. When normalizing for gene length, we observed a similar number of TFBS SVs in ADME genes and drug targets (*p* = 0.52 for Wilcoxon Rank Sum test based on the 1276 non-coding putatively functional SVs) with deletion and insertions being the most common variant types.

**Table 2:** Putative effects of common non-coding structural variants in ADME genes.

Tissue	Gene	Type	Carrier frequency	Affected transcription factors	Potential effect	PGx evidence
Small intestine	*SLC10A2*	DEL	1 in 2 individuals	CTCF	Docetaxel toxicity	^57^
Stomach, small intestine, colon	*SLC7A5*	DEL, INS	1 in 3 individuals	POLR2A, HNF4A	Gastrointestinal melphalan toxicity	^58^
Kidney, Small intestine	*SLC28A1*	DEL	1 in 3 individuals	CTCF, RAD21, TRIM22	Gemcitabine clearance	^59^
Brain, Stomach, Colon	*SLC16A1*	DEL	1 in 10 individuals	SP1	Valproic acid exposure	
Liver	*SULT2A1*	DUP	1 in 10 individuals	CTCF, CHAMP1, CREB1	Acetaminophen induced birth defect	^60^
Stomach, Small intestine, Colon	*SLC29A1*	DEL	1 in 11 individuals	SP1	Ribavirin exposure	^61^
Liver, Small intestine	*CYP4F11*	INS	1 in 24 individuals	HNRNPK	Warfarin does requirement	^62^
Kidney	*UGT1A6*	DEL	1 in 33 individuals	RXRA, JUND, HNF4A, CEBPB, RAD21, HDAC2, NR3C1	Valproic acid exposure	^63^
Lung, Liver	*GSTP1*	DEL	1 in 44 individuals	HNF4A, RXRA, JUND, FOS, KDM1A, JUN, CEBPB	Cisplatin response	^64^

Note that not all transcription factors affected by the respective SVs are shown. For the comprehensive list, we refer to Supplementary Table 4.

*DEL* deletion, *INS* insertion.

In addition to ADME genes, we also discovered a multitude of SVs that impacted transcription factors co-expressed with drug targets (Table 3). For instance, the upstream region of *GABRP* encoding the π subunit of the GABA_A_ receptor that constitutes the target of a multitude of mostly anesthetic and anxiolytic drugs, contains a frequent insertion polymorphism (MAF = 62.4%) that impacts the TFBS of the neuronal transcription factors MAFK, which could modulate *GABRP* expression in the central and enteric nervous system. Similarly, expression of the prostaglandin receptor *PTGER4* in the lung might be impacted by common deletions of JUND and SP1 binding sites (MAF = 14.2%), which might have important roles in the modulation of prostaglandins in allergic pulmonary inflammation and asthma. These analyses constitute to our knowledge the first systematic evaluation of the impact of structural pharmacogenomic variation on experimentally validated transcription factor binding motifs and will provide an important resource for future biological validation efforts.

**Table 3:** Tissue-specific drug response that might be affected by putatively functional non-coding SVs in drug target genes.

Tissue	Gene	Type	Carrier frequency	Affected transcription factors	Potential effect on	PGx evidence
Brain	*GABRP*	INS	1 per individual	MAFK	Response to propofol	
Brain	*GRIN2A*	DEL	1 in 3 individuals	FOXK2	Ketamine exposure and response	^65^
Colon	*TOP1*	DEL, INS	1 in 13 individuals	GATA3	Irinotecan response	^66^
Adipose tissue	*PPARA*	DEL, INS, CPX	1 in 17 individuals	POLR2A	Fenofibrate response	^67^
Brain	*ABAT*	DEL	1 in 20 individuals	HDAC2	Valproic acid response	^46^
Heart	*PDE3A*	DEL, INS, CPX	1 in 197 individuals	RXRA	Amrinone response	
Brain	*HTR2A*	DEL, INS	1 in 1548 individuals	KDM1A	Response to antidepressents	^48^
Brain	*MTNR1B*	DEL	1 in 1706 individuals	RAD21	Response to tasimelteon	
Brain	*HTR1A*	DEL, INS	1 in 2717 individuals	CTCF	Response to antipsychotics	^68^
Heart	*PDE3A*	DUP	1 in 2717 individuals	CTCF	Amrinone response	
Stomach	*ATP4A*	DUP	1 in 3623 individuals	NR3C1	Omeprazole response	
Brain	*MAOA*	DEL	1 in 3994 individuals	FOS	Isocarboxazid response	
Brain	*MTNR1B*	DUP	1 in 5435 individuals	RAD21	Response to tasimelteon	
Brain	*GABRA1*	DUP	1 in 10870 individuals	RAD21	Response to GABA ligands	^69^
Brain	*GABRA1*	INS	1 in 10870 individuals	ATF2	Diazepam response	^69^

Note that not all transcription factors affected by the respective SVs are shown. For the comprehensive list, we refer to Supplementary Table 4.

*CPX* complex rearrangement, *DEL* deletion, *DUP* duplication, *INS* insertion.

### Impact of SVs on pharmacogene expression

**Fig. 4:**
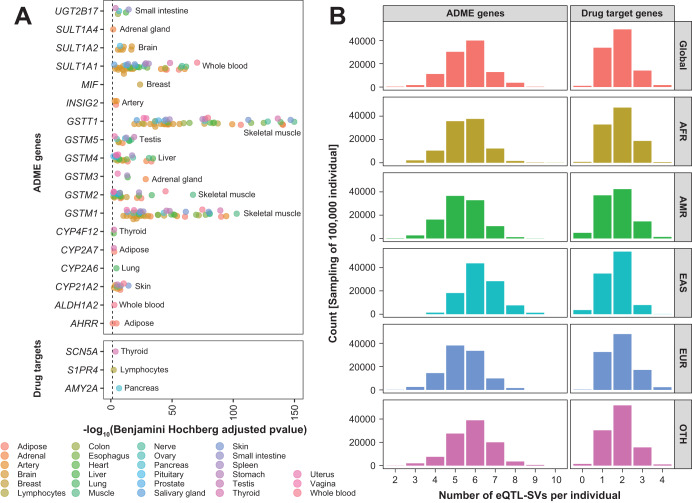
Impact of structural variation on pharmacogene expression. **A** Structural variants in pharmacogenes associated with mRNA expression across multiple GTEx tissues. **B** Distribution of structural variants associated with mRNA expression levels of ADME and drug target genes. Carrier numbers are shown for a simulated population of 100,000 individuals. AFR Africans, EUR Europeans, AMR admixed Americans, EAS East Asians, OTH Others.

To systematically interrogate the functional impact of PGx and drug target SVs, we mapped the profile of pharmacogenomic SVs to published multi-tissue eQTL data from the GTEx project^26^. Because of different detection workflows and cohort sizes between the eQTL study and gnomAD, the number of detected SVs differed more than 7-fold between both studies (approx. 61k to 433k) and only 23% of SVs mapped within 100 bp in both data sets. In total, we found 21 common SVs of ADME and drug targets (15 coding, 6 non-coding) that were significantly associated with mRNA expression (Table 4). As expected, well-known functional SVs of *AMY2A*, *CYP2A6*, and its corresponding pseudogene *CYP2A7*, *CYP21A2*, *GSTM1*, *GSTT1*, *SULT1A1*, and *UGT2B17* are significantly associated with mRNA expression in various tissues (Table 4, Fig. 4A). Of note, *CYP2D6* SVs, which are known to improve phenotypic predictions^27^, are not included in the GTEx dataset, likely due to issues with appropriately calling variations in this complex locus^28^.

**Table 4:** Common eQTL SVs located in ADME genes and drug targets.

Gene	SV type	SV Function	Affected TFBSs	Tissues with eQTL	-log10(BH) [range]	Beta value [range]^a^	Matched SVs^b^	GnomAD AF [range]
*AHRR*	DUP	non-coding	EGR1, NRF1, POLR2A, RNF2	2	1.48, 4.61	0.97, 1.48	0	NA
*ALDH1A2*	DUP	coding		1	2.84	0.97	1	0.08
*AMY2A*	MCNV	coding		1	6.72	0.16	2	0.14–0.19
*CYP2A6*	MCNV	coding		1	4.56	0.53	4	0.01–0.04
*CYP2A7*	MCNV	coding		2	2.46, 2.81	−0.63, −0.53	3	0.01–0.04
*CYP4F12*	DEL	non-coding	MAX, EP300, SP1, POLR2A, REST, JUND, NR3C1, CTCF, HNF4A, FOXA1, HDAC2, FOXA2, RXRA, RAD21, ZNF24	2	2.36	−1.06, −0.84	1	0.02
*CYP21A2*	MCNV	coding		10	2.56, 14.39	−0.44, −0.21	0	NA
*GSTM1*	MCNV	coding		25	10.29, 102.8	0.52, 0.64	1	0.85
*GSTM2*	MCNV	coding		15	2.02, 67.09	0.08, 0.41	1	0.85
*GSTM3*	MCNV	coding		3	5.59, 28.48	0.39, 0.49	0	NA
*GSTM4*	MCNV	coding		15	2.09, 34.44	0.14, 0.46	0	NA
*GSTM5*	MCNV	coding		8	3.14, 19.03	0.17, 0.42	0	NA
*GSTT1*	MCNV	coding		40	19.24, 149.8	0.55, 0.67	1	0.72
*INSIG2*	DEL	non-coding	ATF2, ATF7, CTCF, FOS, POLR2A, STAT3	3	3.13, 4.76	−1.08, −0.9	1	0.03
*MIF*	DEL	non-coding	CTCF, HNF4A, RAD21, RXRA, SMC3, TRIM22	1	23.9	0.82	0	NA
*S1PR4*	DEL	coding		1	2.33	0.51	2	0.82
*SCN5A*	INS	non-coding		1	3.82	−0.38	1	0.31
*SULT1A1*	MCNV	coding		26	2.8, 70.48	0.19, 0.49	1	0.48
*SULT1A2*	MCNV	coding		3	5.8, 17.1	−0.51, −0.32	0	NA
*SULT1A4*	MCNV	coding		1	1.92	0.18	0	NA
*UGT2B17*	DEL	coding		5	3.87, 14.53	−1.2, −0.7	1	0.56

*AF* allele frequency, *eQTL* expression quantitative trait locus, *DUP* duplication, *DEL* deletion, *INS* insertion, *MCNV* multi-copy number variation, *TFBS* Transcription factor binding site, *BH* Benjamini–Hochberg value, *SV* structural variant.

^a^Effect size of the correlation between SV and gene expression. Values greater than 0 reflect increased expression levels.

^b^A matched eQTL variant is defined as a SV of the same type (e.g., DUP, DEL) that was identified in both gnomAD and GTEx.

A very frequent partial deletion within the *S1PR4* locus (combined MAF = 0.64) were significantly correlated with its expression in lymphocytes (Benjamini-Hochberg [BH] *p* < 0.005). This finding is interesting as reduced expression of S1PR4 has been associated with protection from diet-induced non-alcoholic steatohepatitis and hepatic fibrosis^29^. Interestingly, almost one in five individuals carried homozygous *S1PR4* deletions and there was a population difference in SV frequency from 53% in East Asians, Latinos (65%), Africans (88%) to European subjects (90%). Similarly, a previously described intronic deletion (MAF = 2%) of *CYP4F12*, which covers several TFBSs^30^, was associated with decreased expression in thyroid and heart tissue (BH *p* < 0.004). Furthermore, depending on the transcript reference, a 1.2 kb upstream or partial coding duplication of *ALDH1A2* was associated with higher expression in blood, while a non-coding deletion (covering TFBS) of *INSIG2* was associated with decreased expression in adipose and artery tissues.

Overall, each individual carried on average one structural eQTL that impacted the expression of drug targets and 3–5 variations affecting ADME gene expression (Fig. 4B). Interestingly, the distribution of eQTL-SVs per individual were overall similar between Europeans, Africans and admixed Americans, whereas the number of ADME SVs was considerably higher in East Asians. Based on these data, we carefully estimated the functional impact of non-coding structural variations (see Eq. (1) in the Methods section for details). Specifically, by cross-referencing the number of functional non-coding SVs in ADME genes and drug targets (21.7 per individual), as well as the number of functional exonic SVs in ADME genes (10.3 per individual) and drug targets (1.5 per individual) calculated in this study with data about the functional impact of with available information about the number of functional SNVs in ADME genes (40.6 per individual) and drug targets (26 per individual) from the literature^10,31^, we calculated that non-coding structural variants account for approximately 22% of the overall genetically encoded pharmacogenomic variability. As such, both coding and non-coding SVs constitute a considerable source of pharmacogenomic variability, the latter of which is not commonly considered by studies into heritable factors of drug response and safety.

## Discussion

SVs are important mutational forces that shape genomic organization and biological functions^32^. Compared to SNVs, SVs are substantially understudied, at least in part due to the difficulties associated with their identification via commonly used short-read sequencing technologies. While over 500,000 SVs have been described across the human genome^19^, only a small minority of those are functionally understood. In ADME genes, information about structural variability has long been limited to CNVs and complex rearrangements in few selected loci, such as *CYP2A6*, *CYP2D6*, *SULT1A1*, and various *GSTs*^33^. Even less information was available about the structural variability in drug targets where analyses were largely limited to the *AMY1*/*2* locus^34^. While CNVs in other drug target genes, such as *PGA5*, have been described in genome-wide studies^35,36^, their precise architecture and functional effects on drug response have not been analyzed. Building on these findings, we here compiled an overview of the structural pharmacogenomic variome across 908 ADME and drug target genes based on publicly available SV data. These data provide a comprehensive map of structural variability in human pharmacogenes and constitute the basis for the first functional interpretation of both coding and non-coding pharmacogenomic structural variation.

Structural variability is of considerable importance for determining the molecular phenotype of cells with 18% of total detected genetic variation in gene expression being attributed to CNVs^37^. Of all pharmcogenomic SVs identified, 775 (5.2%) were annotated as putatively causing functional consequences (Supplementary Table 1). Examples include common SVs in multiple *CYPs, GSTs* and *UGTs*, as well as in a few drug target genes, primarily those encoding laminins and amylases (Table 1). Furthermore, our data corroborated previous findings of *SULT1A1* duplications^38^, which can translate into enhanced phase II metabolism of multiple drugs (e.g. acetaminophen and tamoxifen) and hormones (e.g. estrogen)^39^. However, the functional consequences of the remaining 14,209 SVs, consisting primarily of those that were located up- and downstream of the gene or that affected UTRs or intronic regions, had not been assigned using current annotation guidelines.

In non-coding regions of the genome, SVs can affect regulatory sequences, such as TFBS, and such variation has been shown to impact gene expression, biological functions and disease risk^40–42^. However, associations of non-coding SVs with drug-related effects have been lacking. We thus integrated structural genomics data with transcription factor binding signatures and expression data across key tissues involved in drug action and drug disposition to pinpoint potential impacts of such non-coding structural variability on drug-related phenotypes. Our analyses identified 1276 SVs that impact experimentally validated TFBS in pharmacogenetic regulatory elements. In ADME genes, multiple common SVs were identified that impact TFBS upstream of the SLC transporters *SLC7A5* (encoding LAT1), *SLC16A1* (MCT1), *SLC28A1* (CNT1), and *SLC29A1* (ENT1), implicated in the disposition of melphalan, valproic acid, gemcitabine or ribavirin, respectively. Notably, while genes encoding *CYP* enzymes or transporters of the SLC and ABC superfamilies have previously been identified as highly variable at the level of single nucleotide polymorphisms^43–45^, these results show that, surprisingly, common structural variants affecting TFBS are predominantly found in *SLC* genes.

Examples of non-coding SVs with putative relevance for drug response include the deletion of a regulatory element upstream of the drug target gene *ABAT* that is found in 1 in 20 individuals. *ABAT* encodes GABA transaminase, one of the key pharmacodynamic targets of valproic acid. While SNVs in *ABAT* had previously been associated with valproic acid response^46^, the impacts of structural variation in this gene have to our knowledge not yet been addressed. Our results suggest that structural variants alter the recruitment of HDAC2, a histone deacetylase expressed in the CNS that controls chromatin accessibility^47^, which in turn might impact *ABAT1* gene expression. Further examples are copy number variants of binding sites for the lysine demethylase KDM1A in the locus encoding the serotonin receptor *HTR2A*. Previous studies suggested that *HTR2A* activity associates with response to antidepressive treatment and remission of depressive symptoms^48^. Moreover, genetic manipulation of lysine methyltransferases in mice was shown to alter *Htr2a* expression and histone methylation has thus been proposed as an epigenetic drug target for anxiety and depression^49^. Our findings thus suggest that structural variability of the *HTR2A* locus might impact epigenetic remodeling and gene expression, thus potentially contributing to serotonergic signaling and response to selective serotonin reuptake inhibitors (SSRIs).

Combined, our results provide the most comprehensive map of coding and non-coding structural variations in the human pharmacogenome published to date. Furthermore, we provide the first functional interpretation of this structural variability, highlight a multitude of structural variants with putative tissue-specific impacts on drug response or toxicity due to deletion or insertion of regulatory elements for further experimental and epidemiological validations. Our data indicate that non-coding structural variants might present an understudied, but important class of variation, which might account for 22% of genetically encoded pharmacogenomic variability. As such, the presented findings constitute an important resource for variant prioritization and incentivize the incorporation of both coding and non-coding pharmacogenomic variability into personalized drug response predictions.

## Methods

### Structural variant analysis

Structural genomic data for 908 pharmacogenes (344 ADME genes and 564 drug targets) from 10,847 unrelated individuals was extracted from gnomAD^19,50^. The ADME genes were selected based on previous work describing a targeted sequencing panel for ADME sequencing^51^. As drug target genes, we considered all genes that encode a target of an FDA-approved drug that was encoded in the nuclear genome^10^. In total 387,477 SVs were identified of which variants with filter status other than “PASS” or “MULTIALLELIC” and type of “unresolved non-reference breakpoint junction” & “reciprocal translocation” were excluded (*n* = 305,149 after this exclusion). SVs with neighboring intervals were aggregated by gene and SV type using the bed_cluster function from the R package valr^52^. Specifically, we used max_dist = 0 to merge of overlapping and directly adjacent intervals, resulting in 256,429 unique SVs genome-wide. Subsequently, we filtered for overlap with the 908 pharmacogenes (Gencode v19), yielding a total of 14,984 SVs across the human pharmacogenome (Supplementary Table 1). SVs spanning more than one pharmacogene were counted for each gene individually. SVs were annotated as coding when they impacted at least one pharmacogenomic exon or as non-coding when the SV affected only intergenic or intronic regions. Non-coding variants were furthermore analyzed for the presence of transcription factor binding sites (TFBS) using the Transcription Factor ChIP-seq Cluster data (338 transcription factors [TFs], 130 cell types) from ENCODE 3^53^. After exclusion of TFBS with peak scores <200 and single study observations (1/1264), 224 TFs were analyzed. SV categories were extracted from the original study^19^ and translated into putative functional consequences according to Supplementary Table 2. Information about 440 olfactory-related genes was extracted from the KEGG pathway “hsa04740”. Tissue-dependent expression levels of candidate genes and TFs were evaluated using median gene-level RNA-Seq data from GTEx^26^. Information about significant associations between SVs and RNA-seq expression was obtained from a multi-tissue eQTL study^54^. The data was filtered for SV-eQTLs, and gene information was added using biomart. The overlap between the breakpoints of SV-eQTLs and gnomAD-SVs was assessed using the bed_closest function from valr^52^. Furthermore, SV-eQTLs that overlapped >99% with gnomAD-SVs were included in the analyses. The carrier frequency or number of total SVs associated with mRNA expression was assessed by simulating 100,000 individual using reported allele frequencies in gnomAD.

### Calculation of the functional impact of non-coding structural variations

The relative functional importance of non-coding SVs was calculated according to Eq. (1) as follows:

1 $${{func}}_{{ncSV}}=\frac{{n}_{{ncSV}}}{{n}_{{ncSV}}+{n}_{{SNV}}+{n}_{{cSV}}}$$

with *n*_ncSV_ defined as the number of functional non-coding SVs in ADME genes and drug targets per individual, *n*_cSV_ defined as the number of functional exonic SVs in ADME genes and drug targets per individual and n_SNV_ defined as the combined number of functional SNVs in ADME genes and drug targets per individual. The number of SNVs in ADME genes per individual was obtained from ref. ^31^, while the number of SNVs in drug target genes was calculated from ref. ^10^ by aggregating all drug target variants with putative functional impacts weighted with the respective frequencies in the entire cohort.

### Statistical analyses

Common variations were defined as variants with a minor allele frequency (MAF) ≥ 1%, while SVs with frequencies <1% were considered as rare. All analyses including the filtering steps were performed using R version 4.0.1 with the additional packages tidyverse_1.3.0^55^, valr_0.6.1^52^, ggsignif_0.6.0^56^. If not other stated, we used Wilcoxon Rank Sum Tests to compare continuous data between groups. All tests were two-sided and significance was assumed at 0.05.

### Reporting summary

Further information on research design is available in the Nature Research Reporting Summary linked to this article.

### Supplementary information

Supplementary Figure 1

Reporting Summary

Supplementary Table 1

Supplementary Table 2

Supplementary Table 3

Supplementary Table 4

## Data Availability

SV data is available via gnomAD (https://gnomad.broadinstitute.org/), TFBS data is provided by ENCODE (https://www.encodeproject.org) and eQTL information is available via the GTEx Portal (https://gtexportal.org/home/). All these repositories are publicly available.
