# Supplementary material for: Structural variation of the coding and non-coding human pharmacogenome
Source: NPJ Genom Med. 2023 Sep 8;8:24. doi: 10.1038/s41525-023-00371-y (PMC10491600; doi:10.1038/s41525-023-00371-y)
Supplement: Supplementary file 1 — Supplementary Figure 1 [file 41525_2023_371_MOESM1_ESM.pdf]

Supplementary Figure 1

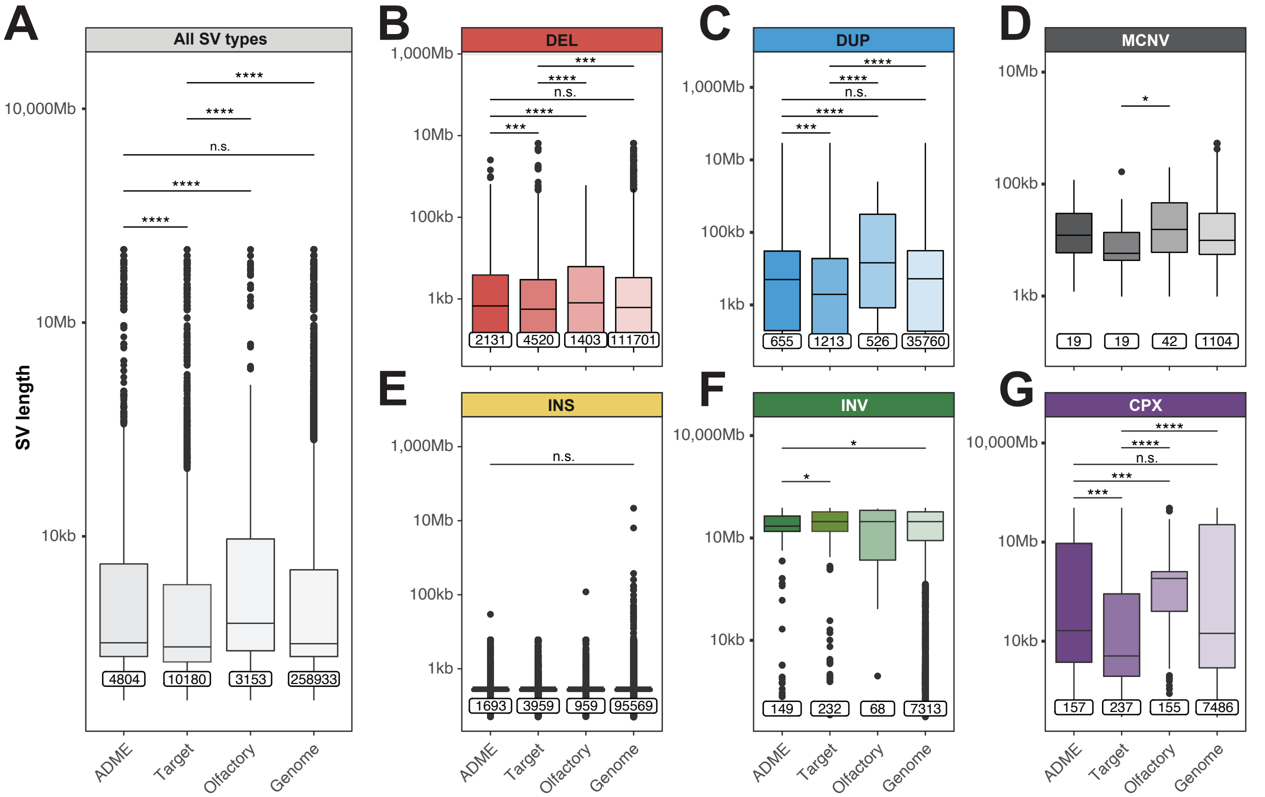

Supplementary Figure 1: Overview of the absolute sizes of structural variations in the human pharmacogenome. Box-and-whisker plots showing the lengths of all structural variations (SVs; **A**) as well as for deletions (DEL; **B**), duplications (DUP; **C**), multi-copy number variations (MCNV; **D**), insertions (INS; **E**), inversions (INV; **F**) and complex rearrangements (CPX; **G**) in ADME genes, drug targets, olfactory genes and across the entire human genome. Numbers under the boxes indicate the total number of SVs identified in the respective category.
